# Supplementary material for: Post-stress changes in the gut microbiome composition in rats with different levels of nervous system excitability
Source: PLoS One. 2023 Dec 11;18(12):e0295709. doi: 10.1371/journal.pone.0295709 (PMC10712864; doi:10.1371/journal.pone.0295709)
Supplement: Attachment — Submitted filename: Response to Reviewers_V2.docx [file pone.0295709.s005.docx]

Thank you for your careful reading of our manuscript and valuable comments.

Below are our answers to each of them.

**Reviewer #2**:

1. My major concern is the difference between the “control” group time 0 and the “before stress” group. Theoretically these two groups should be similar as both groups are not treated yet. However, there are significant differences in Shannon index (Figure 3A)

   Fig 3 - The horizontal axis on panel A shows the animals of the control groups at the corresponding time after the end of stress in the experimental groups. The horizontal axis on panel B shows the intact animals of the consolidated control. As we noted (lines 219-220) we did not find any statistically significant effect of stress on alpha diversity indexes in LT and HT rats (paired t-test).
2. and levels of g. Prevotella and g. Faecalibacterium (Figure 5). The conclusion that HT strain has more diversity than LT strain is not supported if comparing using the “before stress” groups. Can the authors explain the potential differences?

We noted (lines 245-248), that we found that in one case (Fig 5, *g. Prevotella*) the control HT rats significantly differed from the experimental group before stress, which makes it incorrect to compare control groups with experimental ones to assess the effect of stress in this case. But in case of g. Faecalibacterium - there were no significant differences (case/control - unpaired t-test).

We have explained the potential differences of *g. Prevotella* (Lines 317-324)

3. In figure 2, combining data points from HT control group for Chao1 index is questionable as the day 24 is quite different from day 0.

We specifically checked the absence of significant differences in microbiota diversity indices in rats stool of the experimental group before and at different periods after stress. As the paired t-test shows, there are no significant differences. However, the variability of the data is indeed higher in the HT group both in the case of the Ciao1 index and in the case of the Shannon index. This is also in accordance with the assumption of greater dynamism in the fluctuations of microbiota composition in the stool of high-excitable rats compared with low-excitable ones.
